# *Hydraena* (*Hydraenopsis*) *ateneo*, new species (Coleoptera, Hydraenidae) and other aquatic Polyphaga from a small habitat patch in a highly urbanized landscape of Metro Manila, Philippines

**DOI:** 10.3897/zookeys.329.5955

**Published:** 2013-09-05

**Authors:** Hendrik Freitag

**Affiliations:** 1 Ateneo de Manila University, Department of Biology, School of Science & Engineering, Loyola Heights, Quezon City 1101, the Philippines

**Keywords:** Water Beetles, Coleoptera, *Hydraena*, Manila, Luzon, Philippines, taxonomy, urban biodiversity

## Abstract

Seven species of Hydraenidae, Hydrophilidae and Elmidae are recorded from temporary freshwater habitats at the Ateneo de Manila University Campus in the metropolitan area of Manila, Philippines. They were identified as *Enochrus* (*Lumetus*) *fragiloides* d’Orchymont, *Helochares* (*Hydrobaticus*) *lepidus* d’Orchymont, *Helochares* (*Helochares*) *pallens* (MacLeay), *Hydraena* (*Hydraenopsis*) *scabra* d’Orchymont, *Hydraena* (*Hydraenopsis*) *palawanensis* Freitag & Jäch (new record for Luzon Island), *Stenelmis* sp. A further hydraenid species was unknown to science and is newly described: *Hydraena* (*Hydraenopsis*) *ateneo* Freitag, **sp. n.** Aedeagus, gonocoxite, spermatheca, and female tergite X are illustrated by computer-based line drawings. Habitus images of all three *Hydraena* Kugelann species recorded and a checklist of the Philippine *Hydraena* are provided. The presence of these seven species in the Ateneo campus is briefly discussed in regard to the area’s history. Measures to maintain and extend semi-natural islands of biodiversity in urban areas are suggested.

## Introduction

**Figure 1.**
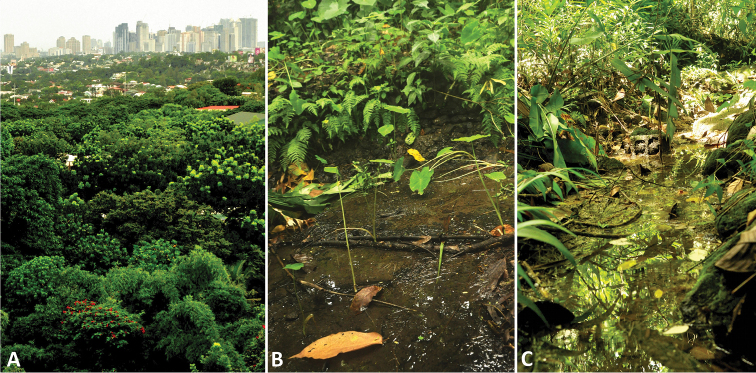
**A** View of Metro Manila from the well-vegetated campus of the Ateneo de Manila University with the collecting sites **B** Temporary pool (label code: ADM2e) where *Stenelmis* sp., *Hydraena scabra*, and*Helochares lepidus* were collected **C** Temporary headwater creek (label code: ADM3d) where all three *Hydraena* spp. were collected.

The National Capital Region (NCR) of the Philippines, known as Metro Manila, had about 12 Mio inhabitants as of 2010 (NSO 2012) and is among the most populous metropolitan regions of the world when the highly urbanized neighboring areas are included. Its largest administrative unit is Quezon City with more than 2.7 Mio inhabitants (NSO 2012), where the study site, the campus of the Ateneo de Manila University, is situated (Fig. 1A).

Semi-natural aquatic habitats within the campus of the university (Figs 1B, C) have been sampled for aquatic insects during a training of Ateneo BSc. Life Science students in 2012. Subsequent identification revealed the presence of three *Hydraena* Kugelann species, of which one was undescribed. This discovery is not surprising as the genus is mega-diverse and the hydraenid fauna of many islands of the Philippines is not yet thoroughly studied. Only Palawan and Busuanga were rather comprehensively studied (Freitag and Jäch 2007). Another survey of the Ateneo de Manila University in Mindoro is currently ongoing.

Only two *Hydraena* species were previously recorded from Luzon: *Hydraena (Hydraenopsis) scabra* d’Orchymont, 1925 and *Hydraena (Hydraenopsis) boettcheri* d’Orchymont, 1932.

Three species of the predominantly aquatic beetle family Hydrophilidae were recorded too. This species-rich family has more than 60 species recorded from the Philippines in about 30 genera (Hansen 1999). Moreover, a single specimen of the family Elmidae was recorded. Less than 30 Elmid species are known from the country so far. They are usually found in lotic freshwater habitats.

## Materials and methods

Three permanently water-filled ponds, five temporary rock pools and three temporary creeks, most of them in small forest-like patches of secondary vegetation, were sampled for aquatic insects. All temporary pools and creeks contained at least some water for most of the time of the year except for a few months (January to May) in the dry season.

The first survey was done at the beginning of the dry season in November 2012 when the water bodies were still filled with water. A replicate sampling was performed in June 2013, the second month of the wet season.

Bottom substrates were physically disturbed in a way that drifted arthropods could be collected by the use of a fine-meshed hand net. Additionally, submerged coarse particulate organic matter, mainly leaves, were manually taken off from the water and checked for benthic organisms. Surprisingly, aquatic beetles were almost exclusively caught in temporary pools and creeks (except for two specimens from a permanent pond). The collection method used might have been less suitably for pond-dwelling beetles. However, a single specimen of an aquatic beetle was caught by an insect light trap near a permanent pond and is also included in this study. Only adults of True Water Beetles (*sensu* Jäch 1998) of the suborder Polyphaga are treated in this taxonomic report.

The material was initially preserved in 95% ethyl alcohol. All type material designated herein and other dissected voucher specimens were glued on entomological paper and pinned. Further material was examined at the Natural History Museum Vienna, Austria (NMW), the Senckenberg Museum of Zoology, Germany (SMTD), and the Zoological Museum of the University Copenhagen, Denmark (ZMU). Previously unidentified *Hydraena* specimens in these collections were checked for the presence of the new taxon. All additional records were designated as types. The same applies for material collected in Cavite (Luzon) in April 2013 during the “Philippine Aquatic Biodiversity Workshop” of the Ateneo de Manila University.

Specimens were examined with the help of an OLYMPUS SZ 61 stereo microscope. Habitus photographs were taken with the same microscope equipped with a digital photo adapter DINO-EYE. Dissected body parts were transferred in lactic acid for one day and then photographed under an OLYMPUS CX21 compound microscope with the same photo adapter. For each illustration, a series of photographs taken at various focus layers was stacked using the “stack” function (habitus) and “pyramid weighted average” function (dissected body parts) of COMBINEZP software (Hadley 2010). The taxonomic key characters were drawn at the computer as vector graphics in Corel Draw 10 with the help of an underlay of the respective stacked images and by comparison with the actual microscopic specimen (e.g. aedeagus, gonocoxite). Biometric measurements were done by the use of a calibrated ocular micrometer.

### Acronyms

ADMU Ateneo de Manila University, Quezon City, Philippines

CFM Collection Hendrik Freitag, Manila, Philippines, currently deposited in ADMU

NMW Natural History Museum Vienna, Austria

PNM Philippine National Museum Manila, Philippines

SMTD Senckenberg Natural History Collections Dresden, Museum of Zoology, Germany

The morphological terminology used herein follows mainly Jäch et al. (2005).

## Data resources

The data underpinning the analysis reported in this paper are deposited at GBIF, the Global Biodiversity Information Facility, http://ipt.pensoft.net/ipt/resource.do?r=polyphaga_ateneo_manila_data

## Results

Updated check list of the Philippine species of Hydraena

1. *Hydraena (Hydraenopsis) ateneo* Freitag, sp. n. (Luzon)

2. *Hydraena (Hydraenopsis) boettcheri* d’Orchymont, 1932 (Luzon)

3. *Hydraena (Hydraenopsis) busuanga* Freitag & Jäch, 2007 (Busuanga)

4. *Hydraena (Hydraenopsis) castanescens* Freitag & Jäch, 2007 (Palawan)

5. *Hydraena (Hydraenopsis) claudia* Freitag & Jäch, 2007 (Palawan)

6. *Hydraena (Hydraenopsis) hosiwergi* Freitag & Jäch, 2007 (Palawan)

7. *Hydraena (Hydraenopsis) jojoorculloi* Freitag & Jäch, 2007 (Palawan)

8. *Hydraena (Hydraenopsis) kodadai* Freitag & Jäch, 2007 (Palawan)

9. *Hydraena (Hydraenopsis) manguao* Freitag & Jäch, 2007 (Palawan)

10. *Hydraena (Hydraenopsis) nielshaggei* Freitag & Jäch, 2007 (Palawan)

11. *Hydraena (Hydraenopsis) palawanensis* Freitag & Jäch, 2007 (Luzon, Mindoro, Palawan)

12. *Hydraena (Hydraenopsis) pseudopalawanensis* Freitag & Jäch, 2007 (Palawan)

13. *Hydraena (Hydraenopsis) scabra* d’Orchymont, 1925 (Bohol, Camiguin, Luzon, Marinduque, Mindanao, Mindoro, Negros, Palawan, Panay, Siargao)

14. *Hydraena (Hydraenopsis) zetteli* Freitag & Jäch, 2007 (Palawan)

### Taxonomy

#### Family Hydraenidae (Minute Moss Beetles)

##### Hydraena (Hydraenopsis) ateneo sp. n.

http://zoobank.org/3A99B262-09F7-43C1-9EA5-A557B2A763E4

http://species-id.net/wiki/Hydraena_ateneo

Figs 2A , 3A–E

###### Etymology.

The species is named for the Ateneo de Manila University, Quezon City, Philippines, in special recognition of the fiftieth anniversary of the university’s Department of Biology. The type locality of this species lies inside the university campus. The epithet is a proper noun in apposition.

###### Type material.

**Holotype** ♂ (PNM): PHIL: Luzon, NCR, Quezon City, Ateneo de Manila Campus; spring creek N of Jesuit Residence, leaf packs; 14°38'29.6"N, 121°04'53.6"E, 62m asl; leg. Vidal, Go & Freitag 16.Nov.2012 (ADM3d)M”, terminal parts of abdomen and aedeagus glued separately. **Paratypes:** 1 ♂, 2 ♀♀ (PNM): same data as holotype; 1 ♂ (CFM): “PHIL.: Luzon, Quezon City, Ateneo de Manila Campus, near San Jose Seminary, temporary headwater creek, leaf litter; 14°38'06.4"N, 121°04'50.2"E, 38m asl.; leg. H. Freitag 28.Jun.2013 (ADM2d)M”; 2 ♂♂, 2 ♀♀ (NMW, SMTD): “leg. Jäch (15) PHILIPPINEN – Luzon Los Banos 24.11.1992 Mt. Makiling 100m”; 1 ♀ (NMW): “leg. Jäch (16) PHILIPPINEN – Luzon W Los Banos 25.11. Rainbow Falls 1992”; 1 ♂, 1 ♀ (CFM): “PHIL.: Luzon, Cavite, Ternate, Lamesang Bato River; hygropetric; sec. forest. 340m asl. 14°14'02"N, 120°38'56"E; coll. R Lagat, M Lagat, JP Bala 16 Apr. 2013 (Ter1j)M”; 5 ♂♂, 3 ♀♀ (CFM): “PHIL.: Luzon, Cavite, Ternate, Lamesang Bato River; littoral pool; sec. forest. 340m asl. 14°14'02"N, 120°38'56"E; coll. Pangantihon 16 Apr. 2013 (Ter1j)M”.

###### Description.

**Figure 2.**
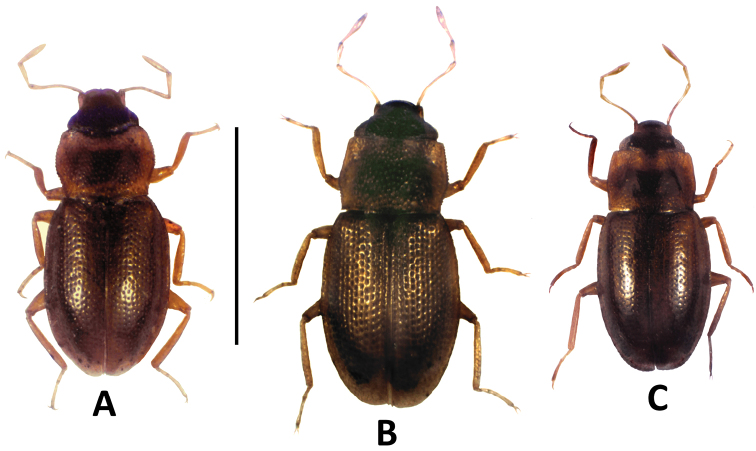
Digital photographs of the habitus of **A** *Hydraena ateneo* sp. n., paratype ♂ **B** *Hydraena scabra* ♀ **C** *Hydraena palawanensis* ♂ (all collected from the Ateneo de Manila University Campus); scale bar = 1.0 mm.

Combined length of pronotum and elytra approximately 1.10–1.14 mm; entire specimen about 1.25–1.33 mm long, 0.58–0.62 mm wide. Dorsal habitus as in Fig. 2A. Elytra brown; pronotum gold-brown with brown sub-rectangular median pattern; frons dark brown; legs, maxillary palpi, and antennae distinctly paler yellowish brown.

Head partly retracted; labrum very slightly excised medially; clypeus moderately densely micropunctate; fronto-clypeal suture slightly concave; frons densely punctate; punctures small and slightly impressed, interstices glabrous; gula rugulose, rest of head venter almost glabrous.

Pronotum about 1.4 times as wide as long, widest at the middle, narrower than elytra; entire pronotum densely punctate; punctures moderately large and moderately deeply impressed, interstices glabrous; anterior margin concave; anterior angles slightly rounded; lateral rim entirely denticulate; lateral margins anteriorly convergent, posteriorly sinuously convergent; pronoto-elytral angle obtuse; posterior margin slightly convex; lateral hypomeron about as wide as profemur. Prosternite carinate, rugulose; mesoventrite with rather indistinct longitudinal ridges, rugulose; mesoventral intercoxal process narrow, distinctly narrower than pseudepipleuron.

Elytra elongately oval, about 1.55 times as long as wide; each elytron with a tiny apical excision next to sutural keel, with about nine, more or less regular rows of punctures between suture and shoulder; punctures moderately large and moderately deeply (apically shallowly) impressed; interstices and intervals glabrous; lateral portion explanate from anterior 0.1 to posterior 0.2; pseudepipleuron anteriorly almost as wide as metafemur, reaching posterior 0.15; epipleuron narrow, short. Metaventral disc slightly impressed; metaventral plaques hardly discernible with stereoscopic microscope, appear as narrow, flat bands; intercoxal segment (abdominal sternite II) slightly wider than long; prosternite, meso-, metaventrite and ventrites 1–4 densely pubescent and rugulose; ventrites 5–6 more or less glabrous, only slightly pubescent.

Femora with almost straight inner margin, outer margin distinctly convex; meso- and metatibiae almost straight, outer faces with few long trichoid setae (sometimes broken off); mesotibia additionally with conspicuous row of short, spine-like setae at inner and outer margin; protibia entirely slightly bent inwards, outer margin slightly convex, inner margin slightly concave to almost straight.

**Figure 3.**
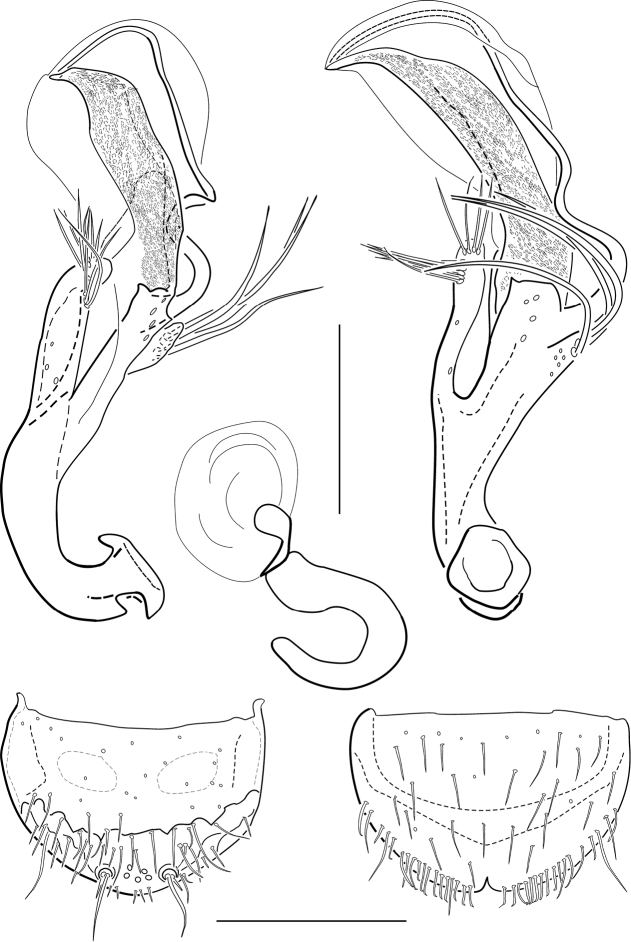
*Hydraena ateneo* sp. n.: **A, B** aedeagus (holotype) in ventral (**A**) and lateral (**B**) view **C** spermatheca **D** gonocoxite **E** female tergite X; scale bars = 0.1 mm.

Aedeagus (Figs 3A, B): Elongate and distinctly bent; main piece more or less straight in ventral view, sinuously curved in lateral view, without setae (some micropores present); phallobase almost symmetrical. Distal lobe long and more or less straight, but bent in 45° angle from main axis, distally tapered, appearing entirely densely freckled or stippled by microstructures; distal lobe embedded in a membranous, transparent, elongate structure. Flagellum very long, bisinuously curved, inserted laterally near right paramere. Parameres firmly fused to main piece; left paramere moderately long and moderately wide, inserted at about basal 0.65 of main piece, latero-apically with approximately five and apically with about four moderately long setae; right paramere extremely short, wider than long; inserted at about basal 0.9 of main piece, with ca. four, almost parallel, long setae.

Gonocoxite (Fig. 3D): Roundly subtrapezoidal, lateral margin slightly convex; setae of subapical tufts straight; margin between apical and basal area uneven, but well developed; apical area moderately densely pubescent; basal area of ventral plate almost without setae; condyles conspicuous; dorsal plate not surpassing outer plate, with two indistinct, separate cavities (hardly discernible in some specimens).

Spermatheca as in Fig. 3C.

###### Secondary sexual characters.

Male profemur with three tiny denticles at basal 0.3 of ventral face (compound microscope recommended). Male terminal sternite subsymmetrically pyriform to elongately subtriangular; base slender, slightly produced laterad; spiculum gastrale almost twice as long as terminal sternite, slightly bent. Male tergite X apically distinctly excised, slightly asymmetrical. Female tergite X (Fig. 3E) subsemicircular; lateral margins evenly rounded; disc moderately densely covered with trichoid setae; setae of subapical fringe short, more or less truncate; each lateral side with one long and several short trichoid setae; hyaline apical margin notched medially.

###### Differential diagnosis.

This species resembles externally *Hydraena castanescens* from which it can be easily distinguished by the smaller size and by its straight metatibiae in both sexes. Furthermore, it can easily be recognized by its aedeagus (distal lobe elongate, freckled, enveloped in a membrane; flagellum very long, bisinuously curved). From the two syntopic species, *Hydraena scabra* and *Hydraena palawanensis*, it can be distinguished by the medium size (1.25–1.33 mm long), the moderately large and moderately deeply impressed elytral punctures, and the shape of aedeagus, gonocoxite and tergite X.

###### Distribution.

So far only known from central Luzon (Quezon City, Cavite, Laguna).

###### Ecology.

This species was collected from small, slow flowing headwater creeks with secondary vegetation cover, where it was found among submerged leaf litter.

##### Hydraena (Hydraenopsis) scabra

d’Orchymont, 1925

http://species-id.net/wiki/Hydraena_scabra

Fig. 2B

Hydraena scabra d’Orchymont 1925: 200 (orig. descr.); Freitag and Jäch 2007 and references therein (redescr., first records from many islands).

###### Material examined.

1 ♂, 1 ♀ (PNM): “PHIL.: Luzon, Quezon City, Ateneo de Manila Campus, near San Jose Seminary, temporary headwater creek, leaf litter; 14°38'06.4"N, 121°04'50.2"E, 39m asl.; 16.Nov.2012, leg. Go, Vidal & Freitag (ADM2d)M”; 3 ♂♂, 1 ♀ (CFM): “PHIL.: Luzon, Quezon City, Ateneo de Manila Campus, near San Jose Seminary, creek side pool, leaf litter; 14°38'05.5"N, 121°04'49.8"E, 37m asl.; leg. Go, Vidal & Freitag 16.Nov.2012 (ADM2e)M”; 1 ♂, 1 ♀ (PNM): “PHIL: Luzon, NCR, Quezon City, Ateneo de Manila Campus; spring creek N of Jesuit Residence, leaf packs; 14°38'29.6"N, 121°04'53.6"E, 62m asl; leg. Vidal, Go & Freitag 16.Nov.2012 (ADM3d)M”; 4 ♂♂, 1 ♀ (CFM): “PHIL: Luzon, NCR, Quezon City, Ateneo de Manila Campus; creek side pool N of Jesuit Residence, subm. leaf litter; 14°38'29.5"N, 121°04'53.6"E, 62m asl; leg. Vidal, Go & Freitag 16.Nov.2012 (ADM3e)M”.

###### Distribution.

*Hydraena scabra* is known from many islands all over the Philippines (see checklist).

###### Ecology.

*Hydraena scabra* is an abundant representative in disturbed lotic water systems, but occurs also in stagnant waters like paddy fields and ponds. On the other hand, this species is often rare or lacking in undisturbed natural sites, where several other species of *Hydraena* can be found syntopically. This suggests a high ecological potency, but low competitiveness.

##### Hydraena (Hydraenopsis) palawanensis

Freitag & Jäch, 2007

http://species-id.net/wiki/Hydraena_palawanensis

Fig. 2C

Hydraena (Hydraenopsis) palawanensis Freitag & Jäch, 2007: 26 (orig. descr.); Freitag and Pangantihon 2010: 140–141.

###### Material examined.

1 ♂ (CFM): “PHIL: Luzon, NCR, Quezon City, Ateneo de Manila Campus; spring creek N of Jesuit Residence, leaf packs; 14°38'29.6"N, 121°04'53.6"E, 62m asl; leg. Vidal, Go & Freitag 16.Nov.2012 (ADM3d)M”; 2 ♂♂, 1 ♀ (PNM): “PHIL.: Luzon, Quezon City, Ateneo de Manila Campus, near San Jose Seminary, temporary headwater creek, leaf litter; 14°38'06.4"N, 121°04'50.2"E, 39m asl.; 16.Nov.2012, leg. Vidal, Go & Freitag (ADM2d)M”.

###### Distribution.

*Hydraena palawanensis* is known from Palawan and Mindoro. It is hereby recorded from Luzon for the first time.

###### Ecology.

This comparatively widely distributed Philippine species occurs in calm zones of undisturbed and moderately disturbed lotic waters. This might indicate a rather high ecological potency. Highest abundances are usually found in the littoral gravel or in residual pools of lowland creeks.

#### Family HYDROPHILIDAE (Water Scavenger Beetles)

##### Enochrus (Lumetus)? fragiloides

d’Orchymont, 1925

http://species-id.net/wiki/Enochrus_fragiloides

Enochrus (Lumetus) fragiloides d’Orchymont 1925: 202 (orig. descr.), 1926: 382; Hansen 1999: 192 (cat.).

###### Material examined.

1 ♀ (CFM): “PHIL: Luzon, NCR, Quezon City, Ateneo de Manila Campus; rock pool near Animal House, subm. leaf litter; 14°38'18.4"N, 121°04'45.7"E, 63m asl; leg. H. Freitag 28.Jun.2013 (ADM5e)M”.

###### Distribution.

This Philippine endemic species is recorded from Palawan and Luzon.

###### Ecology.

As all members of the hydrophilid genera recorded here, this lentic species occurs mostly at the littoral edge of small, calm, temporary water bodies among leaf litter. It probably prefers mesosaprobic waters.

###### Remarks.

This single female cannot be identified with absolute certainty.

##### Helochares (Hydrobaticus) lepidus

d’Orchymont, 1943

http://species-id.net/wiki/Helochares_lepidus

Helochares lentus lepidus d’Orchymont 1943: 5 (orig. descr.).
Helochares lepidus d’Orchymont – Hebauer 1995: 4; Hansen 1999: 168 (cat.) and references therein; Freitag and Pangantihon 2010: 147.

###### Material examined.

2 ♂♂ (CFM): “PHIL: Luzon, NCR, Quezon City, Ateneo de Manila Campus; spring creek near JM Lucas Infirmary, helocrene, leaf packs; 14°33'16.3"N, 121°04'51.7"E, c.45m asl; leg. H. Freitag 16.Nov.2012 (ADM1d)M”; 2 ♂♂, 2 ♀♀ (CFM): “PHIL.: Luzon, Quezon City, Ateneo de Manila Campus, near San Jose Seminary, creek side pool, leaf litter; 14°38'05.5"N, 121°04'49.8"E, 37m asl.; leg. Vidal, Go & Freitag 16.Nov.2012 (ADM2e)M”; 1 ♂, 3 ♀♀ (CFM): “PHIL.: Luzon, Quezon City, Ateneo de Manila Campus, near San Jose Seminary, creek side pool, leaf litter; 14°38'05.5"N, 121°04'49.8"E, 37m asl.; leg. H. Freitag 28.Jun.2013 (ADM2e)M”; 2 ♂♂ (CFM): “PHIL: Luzon, NCR, Quezon City, Ateneo de Manila Campus; pond near CTC, subm. leaf litter; 14°38'18.5"N, 121°04'33.3"E, c.56m asl; leg. H. Freitag 28.Jun.2013 (ADM4e)M”; 1 ♀ (CFM): “PHIL: Luzon, NCR, Quezon City, Ateneo de Manila Campus; rock pool near Animal House, subm. leaf litter; 14°38'18.4"N, 121°04'45.7"E, 63m asl; leg. Go, Vidal & Freitag 16.Nov.2012 (ADM5e)M”; 1 ♂ (CFM): “PHIL: Luzon, NCR, Quezon City, Ateneo de Manila Campus; rock pool near Animal House, subm. leaf litter; 14°38'18.4"N, 121°04'45.7"E, 63m asl; leg. H. Freitag 28.Jun.2013 (ADM5e)M”.

###### Distribution.

This species is endemic to the Philippines, where it is widely distributed, but not yet recorded from Palawan.

###### Ecology.

As in *Enochrus (Lumetus) fragiloides*.

##### Helochares (s.str.) pallens

(MacLeay, 1825)

http://species-id.net/wiki/Helochares_%28s.str.%29_pallens

Enhydrus pallens MacLeay 1825: 35 (orig. descr.).
Helochares pallens (MacLeay) – d’Orchymont 1932: 688; Hansen 1999: 162 (cat.); Hendrich et al. 2004: 128 and references therein.

###### Material examined.

1 ♀ (CFM): “Phil.: Luzon, Quezon City, Ateneo de Manila Campus, San Jose Seminary, semi-stagnant pool, mesosaprobic, light trap; 14°38'05.3"N, 121°04'50.2"E, 37m asl.; leg. L. Quilab, D. Raga & H. Freitag 28.Sep.2012 (ADM2)L”.

###### Distribution.

This species is distributed from New Guinea to the Afrotropical Region and the southern Palaearctic (Hansen 1999).

###### Ecology.

As in *Enochrus (Lumetus) fragiloides*.

#### Family ELMIDAE (Riffle Beetles)

##### Stenelmis sp.

###### Material examined.

1 ♀ (CFM): “PHIL.: Luzon, Quezon City, Ateneo de Manila Campus, near San Jose Seminary, creek side pool, leaf litter; 14°38'05.6"N, 121°04'50.2"E, 38m asl.; leg. H. Freitag 28.Jun.2013 (ADM2e)M”.

###### Remarks.

Surprisingly, this specimen was collected from an almost stagnant (feeder rivulet present) and temporary pool. *Stenelmis* species are usually restricted to streams. Although a single female specimen does not allow a proper determination, it is not unlikely that it belongs to a new species as well since only *Stenelmis palawana* Delève is known from the country, while several Philippine *Stenelmis* s.l. are still undescribed (Freitag and Pangantihon 2010; material at NMW, SMTD and CFM).

## Discussion

The Philippines Islands are one of the most species-rich areas in the world, but suffer from an enormous destruction of their natural habitats and a loss of biodiversity at the same time. Several new species are reported every year from the country. They are usually discovered in the last few remaining natural forests and remote mountain ranges. However, this study has shown that even in a megacity new discoveries are possible, as long as remnants of semi-natural vegetation and more or less unpolluted waters are present.

Although it must be assumed that only euryoecious species inhabit such isolated freshwater habitats, the presence of three *Hydraena* species including one new record for Luzon Island (*Hydraena palawanensis*) and one new species (*Hydraena ateneo* sp. n.) in a small habitat patch amidst a highly urbanized landscape is astonishing.

Based on the copious Palawan records available (AQUA Palawana database, unpubl.), *Hydraena scabra* is often the only species of the genus that can be found especially in disturbed habitats (in 40% of the records *Hydraena scabra* was not collected along with any other *Hydraena* species). If it was collected along with just one other species (in 38% of the records), then this second species was *Hydraena palawanensis* in most cases. The highest *Hydraena* species numbers observed syntopically at the same time was five, in all cases without the occurrence of *Hydraena scabra*.

Photographs and verbal descriptions of the Ateneo campus sampling area before the campus was established are available from the Ateneo Archives (Cruz 1959: pp. 68, 112). The unbuilt area was described as “spacious and rolling grasslands” in 1952. This proves that the sampling sites have been entirely deforested in the last century. Such conditions are unlikely to support assemblages of true aquatic beetles as recorded by now. It can be assumed that the three hydraenids have recolonized the campus area after a tree cover has reestablished and the periods when the creeks and pools contained water became longer.

The presence of an elmid in a temporary pool is interpreted as a recent movement into this habitat from other, somewhat distant running waters, e.g. creeks in the foothills of the Sierra Madre in the neighboring Rizal Province. Flight abilities of water beetles, including *Stenelmis* and other Elmidae, are proven when they are caught at light or in isolated temporary water bodies (pers. obs.). This demonstrates the re-colonization abilities of aquatic insects once they reach suitable habitats. However, the pool where the specimen was collected cannot be considered as an enduring habitat for *Stenelmis*, but clean streams in the campus might potentially be.

Nevertheless, this should not lead to the illusion that the dramatic loss of biodiversity can be reversed. A large proportion of endemic insect taxa such as many other *Hydraena* species is closely associated with indigenous forests with a high diversity of tree species and endemic to a single or few islands (Freitag and Jäch 2007; Freitag and Zettel 2013). When these original habitats are lost, a local mass extinction of animal species is the consequence (e.g. Brook et al. 2003).

To be able to support urban biodiversity and the ecosystem services that these organisms provide, and last but not least to conserve places where students and inhabitants can experience, study, and enjoy nature near their places of residence, the following measures are recommended:

– Minimizing the discharge of untreated sewage into surface waters (e.g. by reed bed treatment);
– Cessation of dumping of wastes into water bodies and forested landscape patches; and
– Planting of indigenous tree species.

## Supplementary Material

XML Treatment for Hydraena (Hydraenopsis) ateneo

XML Treatment for Hydraena (Hydraenopsis) scabra

XML Treatment for Hydraena (Hydraenopsis) palawanensis

XML Treatment for Enochrus (Lumetus)? fragiloides

XML Treatment for Helochares (Hydrobaticus) lepidus

XML Treatment for Helochares (s.str.) pallens

XML Treatment for Stenelmis sp.
